# Rubella Virus- and Cytomegalovirus-Associated Anterior Uveitis: Clinical Findings and How They Relate to the Current Fuchs Uveitis Syndrome Classification

**DOI:** 10.3389/fopht.2022.906598

**Published:** 2022-06-27

**Authors:** Anton Yermalitski, Anne Rübsam, Dominika Pohlmann, Sylvia Metzner, Uwe Pleyer

**Affiliations:** ^1^ Charité – Universitätsmedizin Berlin, Corporate Member of Freie Universität Berlin and Humboldt-Universität zu Berlin, Department of Ophthalmology, Berlin, Germany; ^2^ Berlin Institute of Health at Charité – Universitätsmedizin Berlin, Berlin, Germany

**Keywords:** anterior uveitis, cytomegalovirus, Fuchs uveitis syndrome, Goldmann–Witmer coefficient, Posner–Schlossman syndrome, rubella virus

## Abstract

Rubella virus (RV) and cytomegalovirus (CMV) have both been implicated in anterior uveitis (AU). Clinical phenotypes can vary widely among both etiologies, including Fuchs uveitis syndrome (FUS) as a very distinct phenotype that has been associated with both RV and CMV. The Standardization of Uveitis Nomenclature (SUN) Working Group recently updated the classification criteria for FUS as unilateral AU, including either heterochromia or diffuse iris atrophy combined with stellate keratic precipitates as key findings. The aim of this study was to determine whether our patients adhere to the classification criteria of FUS as previously reported and whether RV- or CMV-associated uveitis can be differentiated by clinical findings. Therefore, this study investigated the clinical characteristics of patients with AU and intraocular presence of either RV or CMV determined by the Goldmann–Witmer coefficient (GWC). Our study included 100 patients (107 eyes) with AU and positive GWC for RV (86) and CMV (21). Clinical findings of RV-positive eyes were as follows: keratic precipitates (91.9%) with a predominantly diffuse distribution (81.4%), unilateral cataract (80.2%), pseudophakia (73.5%), and vitreous cells (59.7%), whereas heterochromia was present in only 39.5% of eyes and iris atrophy in 12.9% of eyes. In CMV-positive eyes, conversely, a higher incidence of ocular hypertension with markedly increased intraocular pressures above 30 mmHg (66.7%), keratic precipitates (81.0%), which were most commonly distributed in the center of the cornea (63.6%), an unaffected lens (55.0%), absent iris atrophy (100%), and absent posterior synechiae (90.5%) could be detected. This indicates a clinical presentation that was mainly compatible with Posner–Schlossman syndrome. In our cohort of RV-positive FUS patients, we saw a different cluster of clinical findings compared to the classification criteria suggested by the SUN Working Group. The main criteria, such as unilaterality, were mostly fulfilled. When applying all classification criteria, only 8.4% of 107 eyes and 10.5% of all 86 RV-positive eyes would qualify for the diagnosis of FUS. In addition, in our cohort of predominantly Caucasian patients, the clinical findings in patients with proven CMV infection differed from the clinical presentation typically associated with FUS.

## Introduction

Uveitis commonly encompasses a broad spectrum of disorders. Only very few entities have been differentiated from each other as early as Fuchs uveitis syndrome (FUS). Indeed, FUS is one of the few uveitic entities that have been considered as a strong phenotype. Typical findings include a white eye with stellate corneal precipitates that are dispersed over the entire corneal endothelium, iris stromal atrophy eventually leading to heterochromia, minimal flare, cells in the anterior chamber, anterior vitreous involvement, and the absence of posterior synechiae. Ernst Fuchs first established FUS, also known as “Fuchs heterochromic iridocyclitis“, in 1906 based on his study of 38 patients (1). While heterochromia was chosen as the “signature phenotype”, several other features such as cataract, absence of synechia, and raised intraocular pressure (IOP) were described as associated with this “syndrome”.

Even though FUS is one of the most common types of anterior uveitis (AU), accounting for up to 22.7% of cases, it is often only diagnosed late because of its insidious onset and undulating clinical course (2, 3). The absence of external signs of inflammation and often subtle clinical features may delay the diagnosis for years and even decades (4). Ophthalmologists are often consulted late, when visual impairment is already present due to vitreous opacification, cataract formation, or glaucoma. Due to variable clinical signs, FUS is often confused with other uveitis entities. As a result, FUS patients are often “over” treated unsuccessfully with topical and systemic steroids or even immunosuppressants (5, 6). While steroids are useful in many other types of uveitis, in FUS, they often enhance cataract formation and further increase progression of glaucoma.

The pathophysiology of FUS has remained an enigma to date. Two decades ago, Quentin et al. documented intraocular synthesized antibodies against the rubella virus (RV) in eyes with FUS (7). RV-associated AU has been described in both unvaccinated and vaccinated young patients; however, an epidemiologic study showed that FUS is less common in patients born since the implementation of the rubella vaccination program in the United States in 1969 (8). Many studies confirmed the relationship between FUS and intraocular rubella antibody production (9–11), however the polymerase chain reaction (PCR) assay is typically negative (9). More recently, deep sequencing detected ribonucleic acid (RNA) in the aqueous humor of FUS patients, supporting the hypothesis of an ongoing viral replication (12). Interestingly, studies from Asia suggested another viral etiology and associated FUS with cytomegalovirus (CMV) infection (13). Hwang et al. previously described two different characteristic clinical profiles of CMV-associated anterior segment infection proven by PCR: profile 1: corticosteroid-resistant inflammatory ocular hypertensive syndrome, and profile 2: corneal endotheliitis with specific coin-shaped keratic precipitates. From profile 1, 16 of 19 patients had an initial diagnosis of Posner–Schlossman syndrome, while this was applicable for only three of 11 patients of profile 2 (14). A possible association of CMV and Posner–Schlossman syndrome has also been described earlier (15).

Identification of an underlying viral etiology may have direct therapeutic implications. While CMV-associated AU responds to Ganciclovir, there is no causal therapy for RV-associated AU (16, 17). The question of whether RV- and CMV-associated FUS can be distinguished by their phenotype has not been profoundly addressed so far in relation to the recent Standardization of Uveitis Nomenclature (SUN) classification.

In 2021, the SUN Working Group established the current international classification of FUS (18). The criteria were determined by a machine-learning algorithm, based on typical clinical findings in patients with FUS with a sensitivity rate of 96.7% and a misclassification rate of 5.5%. Overall, heterochromia or stellate keratic precipitates combined with diffuse iris atrophy were the main clinical characteristics in patients with unilateral AU with or without vitritis. Of note, in clinical practice, however, not all patients show this typical pattern of iris or corneal involvement, and depending on the type of virus responsible (either RV or CMV), clinical findings can differ from the initial description by Ernst Fuchs.

Thus, our work aimed to investigate if there are typical clinical findings in patients with FUS that allow to differentiate FUS according to the causative virus (either CMV or RV). Furthermore, we compared the incidence of clinical findings in our patient cohort with positive RV antibody findings with the clinical criteria established for FUS within the current SUN classification.

## Methods

### Patient Characteristics

We performed a retrospective cohort analysis of clinical records of all consecutive patients, who were seen between January 2000 and April 2013 at the Department of Ophthalmology, Charité Universitätsmedizin Berlin, with AU and a proof of intraocular antibody synthesis against RV or CMV in the aqueous humor. An aqueous humor analysis was performed in all individuals with unclear AU and in all individuals with suspected FUS. Patients who developed AU in the fellow eye during the follow-up underwent an aqueous humor tap on this eye as well, to rule out potential other infectious causes for AU. The study followed the ethical standards of the Declaration of Helsinki and was approved (EA4/075/17) by the Ethics Committee of Charité Universitätsmedizin Berlin.

To exclude the association to any other underlying disorder, patients with a known positive status of human immunodeficiency virus (HIV), syphilis, HLA-B27 genotype, multiple sclerosis, sarcoidosis, tuberculosis, or borreliosis were excluded. The following clinical data were extracted from medical records: sex, age, and the result of aqueous humor analysis (intraocular antibody synthesis against RV and CMV).

To investigate the potential association of RV and CMV, we strictly adhered to the classification criteria for FUS as outlined by the SUN Working Group that defines FUS with the following criteria (18):

1. Evidence of AU
a. anterior chamber cells
b. if vitreous cells are present, anterior chamber inflammation should also be present
c. no evidence of active retinitis

AND

2. Unilateral uveitis

AND

3. Evidence of FUS
a. heterochromia OR
b. unilateral diffuse iris atrophy AND stellate keratic precipitates

AND

4. Neither endotheliitis nor nodular coin-shaped endothelial lesions

Exclusions

1. Positive serology for syphilis using a treponemal test
2. Evidence of sarcoidosis (either bilateral hilar adenopathy on chest imaging or tissue biopsy demonstrating non-caseating granulomata)
3. Aqueous specimen PCR positive for CMV, herpes simplex virus (HSV), or varicella zoster virus (VZV)

In addition, we extended our observations and also included the clinical course: the presence/absence of conjunctival injection, corneal edema, iris nodules, alteration of the lens (presence of cataract or already pseudophakia), type of cataract—if present, presence of macular edema, presence of retinal scars, and markedly elevated intraocular pressure (defined as > 30 mmHg).

### Intraocular Antibody Synthesis

For intraocular antibody analysis, approximately 100 µl of aqueous humor was taken under local anesthesia under aseptic conditions with a 30-gauge insulin syringe under a surgical microscope. Blood samples were collected simultaneously. Blood samples were then centrifuged and stored at +4°C and aqueous humor samples were stored at −20°C until they were processed. The samples were then processed in batches. For the ELISA, 5 µl of serum and 50–100 µl of aqueous humor samples were used. A modified micro-ELISA technique (Enzygnost^®^, Dade Behring Marburg, Germany) was used to detect antibodies in serum and aqueous humor, diluted to an immunoglobulin G (IgG) level of 1 mg/dl according to the manufacturer’s protocol. A comparison of photometric signals (Δ*E* > 0.100) allows the detection of a localized intraocular IgG production against RV and CMV antigens (9). The Goldmann–Witmer coefficient (GWC) was calculated for RV as (anti-RV IgG in aqueous humor/total IgG in aqueous humor)/(anti-RV IgG in serum/total IgG in serum) and for CMV as (anti-CMV IgG in aqueous humor/total IgG in aqueous humor)/(anti-CMV IgG in serum/total IgG in serum). A value ≥ 3.0 was considered positive (19, 20).

### Statistical Analysis

The data were reported as mean ± standard deviation (SD) for normally distributed values or median with interquartile range (IQR) in case of a non-Gaussian distribution. The data were analyzed using the Chi-squared test, the Fisher exact test, and the Mann–Whitney U test where applicable, with IBM SPSS Statistics Version 20. A *p*-value < 0.05 was considered as significant.

## Results

Among the 107 eyes of 100 patients, 86 were positive for a local intraocular antibody synthesis against RV and 21 against CMV. In the RV group, 11 patients (17 eyes) showed bilateral affection. In six of these patients (12 eyes), we were able to follow the clinical course of both eyes, whereas in five patients (five eyes), only one eye was documented. In the CMV-positive cohort, one patient was affected bilaterally, and the clinical records were available for both eyes. In both groups, female patients were slightly more often affected, with 45 eyes (52.3%) in the RV group and 13 eyes (61.9%) in the CMV group (*p* = 0.430). The median age at the time of the anterior chamber tap was similar in both groups (*p* = 0.856) with a median age of 43.9 years (IQR = between 31.9 and 51.8 years) in RV-positive patients and a median age of 41.3 years (27.7–64.5 years) in CMV-positive patients. The demographic data are summarized in **Table 1**. At the time of investigation, none of the 100 individuals presented signs or symptoms of an underlying systemic inflammatory disorder.

**Table 1:** Demographic data of patients with rubella virus (RV) and cytomegalovirus (CMV) positive eyes.

Demographic data	RV-positive eye		CMV-positive eye		*p*-value
Number of eyes	*n* = 86		*n* = 21		
**Median age (IQR)**	43.9 years (31.9–51.8 years)		41.3 years (27.7–64.5 years)		0.856 †
**Sex**	Male: 41 (47.7%)	Female: 45 (52.3%)	Male: 8 (38.1%)	Female: 13 (61.9%)	0.430**

IQR = interquartile range † Mann–Whitney U test ** Chi-squared test

The frequencies of clinical findings are outlined in **Tables 2**, **3**. Based on the five SUN classifications criteria, the following findings emerged: All patients had evidence of AU as this criterion was essential for inclusion in our study. However, at the time of presentation, not all eyes had active AU with anterior chamber cells (no cells: *n* = 60, 56.1%). This applied to both RV-positive and CMV-positive eyes. Vitreous cells were almost exclusively seen in RV-positive patients (*n* = 46, 59.7%), and this differed significantly from our CMV-positive eyes (*n* = 1, 6.7%). There was no evidence of retinitis in either group.

**Table 2:** Absolute and relative frequencies of clinical features of rubella virus (RV) and cytomegalovirus (CMV)-positive eyes grouped according to the Standardization of Uveitis Nomenclature (SUN) Working Group classification.

Clinical feature	RV-positive eye				CMV-positive eye				*p*-value
Absolute and relative frequencies	*n*	%	*n*	%	*n*	%	*n*	%	
**Criterion 1: Evidence of anterior uveitis**									
**Anterior chamber cells**	Positive		Negative		Positive		Negative		
	38	44.7%	47	55.3%	8	38.1%	13	61.9%	0.584**
**Vitreous cells**	Positive		Negative		Positive		Negative		
	46	59.7%	31	40.3%	1	6.7%	14	93.3%	<0.001**
**Evidence of active retinitis**	Positive		Negative		Positive		Negative		
	0	0.0%	86	100.0%	0	0.0%	21	100.0%	1.000*
**Criterion 2: Laterality**									
	Unilateral		Bilateral		Unilateral		Bilateral		
	69	80.2%	17	19.8%	19	90.5%	2	9.5%	0.036**
**Criterion 3: Evidence of Fuchs uveitis syndrome**									
**Heterochromia**	Positive		Negative		Positive		Negative		
	32	39.5%	49	60.5%	0	0.0%	21	100.0%	0.001**
**Iris atrophy**	Positive		Negative		Positive		Negative		
	11	12.9%	74	87.1%	0	0.0%	21	100.0%	0.116*
**Keratic precipitates**	Positive		Negative		Positive		Negative		
	79	91.9%	7	8.1%	17	81.0%	4	19.0%	0.221*
**Criterion 4: Endotheliitis**									
	Positive		Negative		Positive		Negative		
	0	0.0%	86	100.0%	0	0.0%	21	100.0%	1.000*
**Criterion 5: Exclusions** 1. Positive serology for syphilis using a treponemal test 2. Evidence of sarcoidosis (either bilateral hilar adenopathy on chest imaging or tissue biopsy demonstrating non-caseating granulomata) 3. Aqueous specimen PCR positive for CMV, herpes simplex virus (HSV), or varicella zoster virus (VZV)									
	Positive		Negative		Positive		Negative		
	0	0.0%	86	100.0%	0	0.0%	21	100.0%	1.000*

p-values, calculation: * Fisher exact test, ** Chi-squared test.

**Table 3:** Absolute and relative frequencies of clinical features of rubella virus (RV) and cytomegalovirus (CMV) positive eyes.

Clinical feature	RV-positive eye				CMV-positive eye				*p*-value
Absolute and relative frequencies	*n*	%	*n*	%	*n*	%	*n*	%	
**Conjunctival redness**	Positive		Negative		Positive		Negative		
	30	36.6%	52	63.4%	13	61.9%	8	38.1%	0.036**
**Distribution pattern of keratic precipitates**	Diffuse		Localized		Diffuse		Localized		
	48	81.4%	11	18.6%	4	36.4%	7	63.6%	0.004*
**Posterior synechiae**	Positive		Negative		Positive		Negative		
	5	5.8%	81	94.2%	2	9.5%	19	90.5%	0.621*
**Cataract or pseudophakia**	Present		Absent		Present		Absent		
	61	73.5%	22	26.5%	9	45.0%	11	55.0%	0.014**
**Type of cataract**	Posterior subcapsular		Other		Posterior subcapsular		Other		
	30	83.3%	6	16.7%	0	0.0%	9	100.0%	<0.001*
**Vitreous haze > 0**	Positive		Negative		Positive		Negative		
	28	35.4%	51	64.6%	1	6.7%	14	93.3%	0.032*
**Intraocular pressure > 30 mmHg**	Positive		Negative		Positive		Negative		
	16	20.0%	64	80.0%	14	66.7%	7	33.3%	<0.001**

p-values, calculation: * Fisher exact test, ** Chi-squared test.

These findings were not included in the classification of the Standardization of Uveitis Nomenclature (SUN) Working Group for Fuchs uveitis syndrome.

The criterion of unilateral involvement was met with 80% (*n* = 69) for RV and 91% (*n* = 19) for CMV-positive eyes, respectively. Nevertheless, it has to be emphasized that bilateral manifestations were present in 11 RV-positive individuals, whereas in our CMV-positive cohort, only one patient was bilaterally affected.

Heterochromia was not evident in any CMV-positive eye, while it was present in 32 of the RV-positive eyes (39.5%), a feature that highly significantly differed (*p* = 0.001) between the two groups.

Exclusion of corneal endotheliitis or coin-shaped endothelial lesions are required in the fourth SUN classification criterion. This was not evident in any of our RV-positive eyes, while focal precipitates were present in seven CMV-positive eyes (*p* = 0.004). As already mentioned, none of our patients had a systemic infection (syphilis) or sarcoidosis; thus, all patients met the last SUN criterion.

In addition to the FUS-defining features of the SUN classification, we included further clinical findings in our analysis. A substantial intraocular hypertension, defined as an IOP exceeding 30 mmHg, was detected in both groups. In our series of 86 RV-positive eyes, 16 (20%) presented with increased IOP, ranging up to 58 mmHg. However, one of the key findings in our CMV-positive eyes was that seven eyes (66.7%, *p* < 0.001) revealed intraocular pressure peaks of up to 60 mmHg. Cataract formation is also a frequent finding in FUS, leading to impaired vision and early surgical intervention. When we analyzed our data, cataract formation or pseudophakia was present in 61 RV-positive eyes (73.5%) and thus differed significantly from CMV-positive patients: nine eyes (45.0%, *p* = 0.014). Interestingly, in almost all RV cataract eyes (*n* = 30, 83.3%), lens opacity was documented as a posterior subcapsular type. This observation most likely points to an iatrogenic, steroid-induced cataract. In contrast, CMV-positive eyes presented with various types of lens opacification; however, a posterior subcapsular cataract was never documented (*p* < 0.001).

If we now summarize our findings and analyze the diagnostic accuracy of the SUN classification criteria for FUS based on our patient cohort, the following results emerge: Criterion one: “evidence” of AU was present in all patients, whereas criterion two: “unilateral uveitis” was fulfilled by 69 eyes of the RV group (80.2%) and 19 eyes of the CMV group (90.5%). Criterion three: “evidence of FUS” was met by 34 eyes of the RV group (39.5%) and none of the eyes of the CMV group. Decisive for the latter observation is that iris atrophy did not occur in any CMV-positive eye. Criteria four and five: “no endotheliitis” and none of the “exclusion criteria” were met in both groups. Altogether, at least two criteria were accomplished by all eyes of both groups. At least three criteria were fulfilled by 81 eyes of the RV group (94.2%) and 19 eyes of the CMV group (90.5%). At least four criteria were met by 51 eyes of the RV group (59.3%) and eight eyes of the CMV group (38.1%).

**Figure 1:**
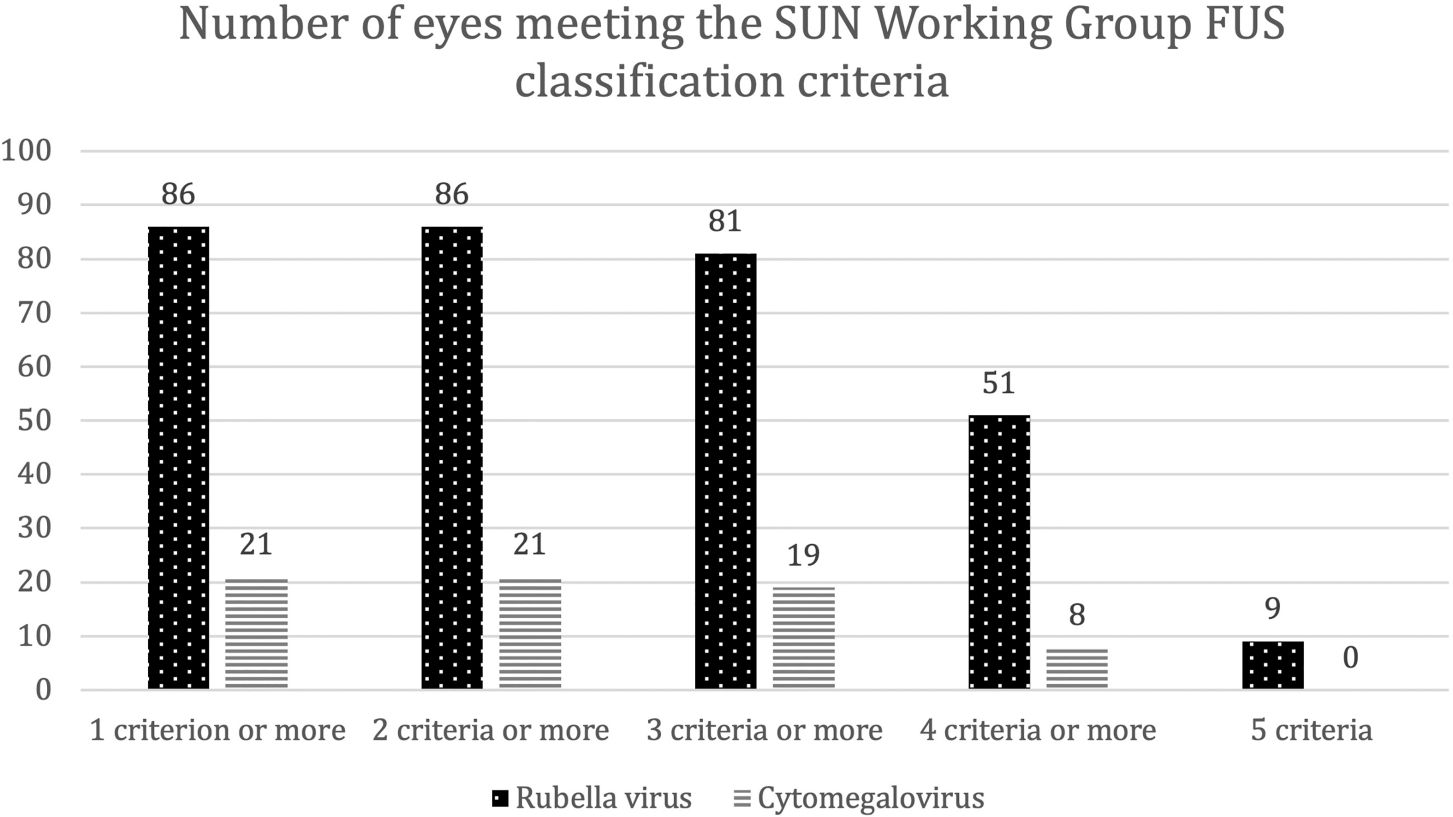
Number of eyes that met the SUN Working Group criteria for Fuchs uveitis syndrome (FUS). Bar chart depicting the numbers of eyes that fulfilled one to five diagnostic criteria for FUS according to the SUN Working Group. This includes **(1)** evidence of anterior uveitis, **(2)** unilateral uveitis, **(3)** evidence of FUS, **(4)** neither endotheliitis nor nodular and coin-shaped endothelial lesions, and **(5)** no exclusion criteria. Eyes are assigned according to the presence of intraocular antibody synthesis against either rubella virus (dotted bars) or cytomegalovirus (striped bars). SUN = Standardization of Uveitis Nomenclature.

All five criteria were only fulfilled by nine eyes from the RV group (10.5%) and none of the CMV group (**Figure 1**).

Thus, only nine patients from our study cohort (all RV-positive) would have been diagnosed as FUS, based on the current SUN Working Group classification criteria.

## Discussion

The SUN Working Group recently developed classification criteria for 25 of the most common uveitic entities including FUS. In a stepwise procedure, 249 cases were collected, of which 146 were used for a database that subsequently underwent a machine learning process. The authors state that the intention was to develop classification criteria with a high specificity rather than diagnostic criteria, which focuses more on sensitivity. The algorithm determined a cluster of clinical findings to detect FUS with a low rate of misclassification (5.5% in the validation set) (18). The stringent combination of clinical findings that are grouped into five different criteria of the current SUN classification may affect clinical practice. Even if it is emphasized that the classification is primarily intended for research and studies, it can be expected that clinicians will also use this classification for diagnostic purposes. Therefore, we applied the current criteria to a cohort of RV/CMV-positive eyes, since both viruses have been previously associated with FUS. In particular, RV has been strongly associated with this “syndrome”.

Based on the current SUN criteria, only nine of 107 (8.4%) eyes or nine eyes of our 86 (10.5%) RV-positive eyes met the FUS classification. This is related to several findings such as the absence of active AU, bilaterality, and iris changes.

The first criterion was only met by a part of our RV-positive patients. However, it must be emphasized that especially in chronic intraocular inflammation, AU does not always reveal anterior chamber cells. Instead, subtle findings such as stellate or medium-sized keratic precipitates or flare in the aqueous are often the only hints. The SUN Working Group defined AU by “the presence of anterior chamber cells and if vitreous cells are present, anterior chamber cells also have to be found”. This strict definition results in a lower sensitivity, since we judged the AU criterion as fulfilled even when anterior chamber cells were absent. The second SUN criterion must also be viewed with caution. In our cohort, 11 of 80 RV-positive patients presented with bilateral uveitis. Indeed, bilateral FUS is not uncommon, affecting about 5%–10% of patients in previous cohorts (4, 10, 17). In addition, the third criterion (“Evidence for Fuchs uveitis Syndrome”) was not met by the majority of our RV-positive patients. Heterochromia as the index finding was present in 39.5% in this cohort. Even when this contradicts the original definition of E. Fuchs, this has also been frequently underlined in previous studies (21, 22). However, heterochromia can be very subtle and might be easily underreported especially in retrospective studies. Besides heterochromia, unilateral diffuse iris atrophy AND stellate keratic precipitates have been defined as evidence of FUS in this SUN classification criterion. In fact, the precipitates in FUS are characteristic and differ from other forms of inflammation. In contrast to other types of AU, keratic precipitates in FUS are often also adherent in the upper corneal endothelium (17). Therefore, this might be a distinguishing feature that may raise suspicion of a FUS. However, the association between iris atrophy and endothelial precipitates was present in only 2.8% of our RV-positive eyes. In contrast, none of our RV patients presented with corneal endotheliitis or nodular, coin-shaped endothelial lesions. Therefore, all patients fulfilled this fourth SUN classification criterion. The exclusion of other etiologies, required as the last criterion, was given in all of our patients, since they underwent our routine diagnostic work-up, including syphilis test and exclusion of sarcoidosis. However, the absence of PCR testing for herpes viruses might mislead to the conclusion that this criterion is not fulfilled. The SUN Working Group stated that “the absence of such testing does not exclude the diagnosis of FUS if the criteria for the diagnosis are met” (18). A PCR confirmation for CMV, HSV, or VZV was not obtained. Instead, we based our diagnosis on intraocular antibody detection. There are various arguments for this. First, and most important, due to the very low sensitivity of RV detection, the infectious etiology would have been compromised. It has been repeatedly confirmed that PCR detection of RV RNA is rare and unreliable in contrast to positive GWC findings (7, 23). Second, to avoid any methodological inconsistency and using the same diagnostic techniques, we applied the intraocular antibody detection to all our patients. The absence of retinitis is also a FUS-defining criterion by the SUN Working Group. In our cohort, none of the eyes showed signs of retinitis. Therefore, we could have omitted this finding, but strictly adhered to the current definition and included this in the *Results* section.

While initial studies associated FUS mainly with RV and were performed in Caucasian patients, later studies also detected CMV as a potential etiology (10, 24). Most of these observations considered CMV as causative for FUS derived from Asia (13, 25, 26). Indeed, according to the current SUN classification, the first two criteria of clinical findings are also met in all of our CMV-positive patients. However, there is a constellation of clinical features that more closely define a CMV-positive Posner–Schlossman syndrome (27). Furthermore, there are significant differences in FUS-defining criteria. None of our CMV-positive patients presented with heterochromia. Likewise, no CMV-positive patient showed unilateral AU combined with iris atrophy and stellate keratic precipitates as required in FUS. In contrast, focal nodular keratic precipitates were evident in several eyes. In addition, markedly raised IOP well above 30 mmHg as a hallmark of Posner–Schlossman syndrome was present in the majority of our CMV-positive patients. Consequently, the SUN Working Group considers a CMV-positive FUS as a separate disease and names it “Fuchs-like” AU (18). Other authors also reported clinical findings in CMV-related FUS that differ from RV-positive FUS (26). In the case of borderline constellations or findings that do not allow a clear diagnosis, aqueous humor analysis remains important, as the underlying infectious etiology may have therapeutic consequences.

Other approaches to diagnose FUS have been suggested. Based on iris imaging combined with artificial intelligence, a recent study considered diffuse iris depigmentation as the most sensitive and reliable sign of FUS (17). This might be an interesting approach given the often-subtle iris changes in particular in dark brown eyes that can easily be overlooked in everyday routine. In particular, bilateral involvement and other subtle changes as small iris nodules might be registered easier with the use of modern technology (28). Besides heterochromia, one of the most common iris changes in FUS is atrophy, which occurs in approximately 30% (29). Other authors reported an incidence of iris changes in FUS in almost 70% of the eyes in a similar population (30). This large variety leads to the presumption that, sometimes, iris changes might be underreported due to the discreet appearance. Since high-resolution slit lamp photography and compact powerful computers are widely available nowadays, more studies are needed to evaluate its use in the diagnosis of FUS. However, since it affects only one of the various clinical criteria, it remains doubtful whether this alone can secure the diagnosis. Instead, it might be a useful screening procedure.

Artificial intelligence was also used in the current SUN classification. Even though this methodological approach appears rational and justifiable, it must also be viewed with care. This current FUS classification is based on a limited sample size with 146 selected data sets used for the consecutive machine learning process (18). Some criteria have been introduced, which do not completely match the current clinical experience, e.g., the absence of active AU, bilateral occurrence, and iris changes. Therefore, the risk of a “self-fulfilling prophecy” may arise. Whether this classification adequately supports the clinical demand might be investigated in further studies.

Taken together, our results indicate that it seems not possible to differentiate between an RV- and a CMV-associated AU based on clinical findings only, although often these diagnoses seem to be obvious. We further support the thesis of an association of CMV and Posner–Schlossman syndrome (31). Therefore, analyzing aqueous humor may allow a more precise definition of the underlying etiology and confirm the diagnosis. The previous notion that intraocular RV antibodies are present in almost all FUS patients not only provides an interesting clue for its etiology, but also raises the option for securing the diagnosis (9–11).

We are well aware about the limitations of our study. The shortcomings are related to its retrospective nature. Ocular findings were not always complete in all cases due to a prolonged recruitment period. Furthermore, in our cohort, the iris color was not documented in all eyes. Therefore, subtle changes particularly in dark irides may have been underestimated. Still, the majority of our patients were Caucasians with brighter irides, and heterochromia is more likely to be detected. For five patients with bilateral uveitis, the clinical records were available only for one eye. Also, the number of CMV-positive eyes was limited as compared to our RV-positive cohort, which reflects the current situation in Europe (2, 32).

More than 100 years after the first description of FUS, many questions regarding its etiology and pathophysiology are still left. Better diagnostic approaches and tools are needed to achieve a shorter latency between first presentation and the correct diagnosis. Previous investigations indicate that the clinical presentation of FUS is incomplete in many patients. In addition, the clinical course in many individuals suggests that the disease represents a continuum of clinical findings that are arising over time. Even if these cannot be directly attributed to a latent RV infection in all its consequences, this virus obviously plays an important role.

Still, the option to secure the intraocular RV antibody synthesis using GWC is not ubiquitous and readily available, but it can be of great benefit. Since classifications in all fields of medicine show a continuous development, this may also be taken into account in the future for FUS.

## Data Availability Statement

The raw data supporting the conclusions of this article will be made available by the authors, without undue reservation.

## Ethics Statement

The studies involving human participants were reviewed and approved by the Ethics Committee of Charité Universitätsmedizin Berlin (EA4/075/17). Written informed consent for participation was not required for this study in accordance with the national legislation and the institutional requirements.

## Author Contributions

AY and AR are first authors with equal contribution. AY collected the data, performed the statistical and logical analysis, and wrote the introduction and part of the discussion. AR analyzed recent publications, compared our data, and wrote conclusions; furthermore, she improved the translation into English. UP corrected the content, conclusions, added ideas, and led the group through the writing process. DP added ideas and corrected logical incompleteness. SM analyzed the samples taken from the anterior chamber and documented the results. All authors contributed to the article and approved the submitted version.

## Conflict of Interest

The authors declare that the research was conducted in the absence of any commercial or financial relationships that could be construed as a potential conflict of interest.

## Publisher’s Note

All claims expressed in this article are solely those of the authors and do not necessarily represent those of their affiliated organizations, or those of the publisher, the editors and the reviewers. Any product that may be evaluated in this article, or claim that may be made by its manufacturer, is not guaranteed or endorsed by the publisher.
